# Medical Jurisprudence

**Published:** 1826-04

**Authors:** 


### Medical Jurisprudence.

44. *College of Surgeons and its Members.**—The storm which had long been gathering over the temple of Esculapius in Lincoln's Inn-fields, burst, on Saturday evening, the 18th February, in torrents—not of rain, nor perhaps of eloquence, but certainly of unmitigated reproach and vehement crimination. The number assembled on this occasion, has, we think, been greatly exaggerated. We estimated it at from six to eight hundred, at the utmost. It has been magnified by fame (or rather by falsehood) to nearly double that number. The meeting contained a very small proportion of men of age, note, or influence in the profession. The great mass consisted of the younger members of the College, including general practitioners, and a considerable proportion of students, who were not members of any College or corporation whatever.

On the motion of Mr. Tyrrel, Mr. Lawrence was called to the chair under loud applause. We did not hear the beginning of his address, and therefore shall take the substance of it from one of the Newspapers, not vouching for its correctness, since we do not find that the proceedings which followed have been by any means, fairly detailed in the public prints.

Mr. Lawrence observed that, as members of the College of Surgeons, they had met, "in order to devise the best means of remedying the abuses which exist in the management of that institution." He did not consider these abuses as personal grievances merely, but as public ones also—since, to remove impediments that obstruct the advancement of surgical knowledge, must be a matter of national concern. After eulogising the liberality of Government, in presenting the College with the Hunterian Museum, he adverted

* We beg it to be remembered that, in the first three or four pages of this article, we are merely chroniclers of the day. Our own sentiments are broached at page 585, *et seq.*

to the early charters of the College, in which the management was entrusted to a master, assistants, and the *commonalty*, by which last class he conceived to be meant the body of members of the College. The share which the *commonalty* had in the management of College affairs, it was difficult to describe—it was neither here nor there—it appeared to consist of a pretty good number of *negative rights*. They had no voice in the election of the ruling powers—no control over the funds—very limited access to the museum—and, finally, they had lately been favoured with a great *extension* of these negative privileges *(laughter)* in the enactment of a bye-law, which is now well known, and which was probably the efficient cause of the present meeting. We need not here reiterate the bye-law in question, since we have more than once expressed ourselves as unequivocally hostile to its object and operation. Mr. Lawrence took occasion to criticise rather severely the *composition* of this unfortunate edict, which he characterised as contemptible in its diction, and odious in its spirit. Its ostensible object is "to promote *sound* chirurgical knowledge," but, said he, "we find that sound or unsound knowledge depends upon the season of the year in which knowledge is acquired. *Sound* anatomical knowledge is that which is gained in the winter season; *(laughter)* unsound anatomical knowledge is that which is gained in summer time." *Laughter.* It is upon such nice distinctions, he continued, that the great body of the profession, not only in London, but in the country, are disqualified from teaching the knowledge of their science to others. After adverting to the circumstance, that the legitimate lecturers do not profess to give any *winter* courses (their courses being the autumnal and spring—which, by the way, is rather a quibble) Mr. L. contended that summer courses of anatomy were very convenient and useful for those who had a multiplicity of prelections to attend in the winter. He then came to the most objectionable part, that of restricting the clinical studies of pupils to certain hospitals, as of London, Dublin, &c. thus excluding many other excellent schools of instruction, as of Liverpool, Leeds, Manchester, Bristol, and several more that could be named:—with which is coupled the equally objectionable restriction of tuition itself, to those persons only who belong to the said favoured hospitals, or who are recognized or acknowledged by them. Upon each of these clauses Mr. Lawrence passed many severe, and we are sorry to say, some *just* strictures. Among other observations, he averred that the College had already violated its own law; for Mr. Kiernan, a gentleman well qualified, had obtained the signatures of the surgeons of St. Bartholomew's Hospital, as to his fitness for teaching anatomy, and yet he was refused a licence by the men in power. "This universal proscription, observed Mr. L. cast a most unmerited stigma on the very eminent men who have the principal care of the provincial hospitals." He then adverted to names that grace the annals of modern surgery, as Hey, Hodgson, Martineau, Dalrymple, Cross, Barnes, &c. &c. remarking—and truly remarking, that the edict was no less injurious to the eminent men thus excluded than to the students themselves—and he might have added, to the community at large, whose interests are involved in the qualifications of those who are to have the charge of their health.

The museum next came under Mr. Lawrence's notice. In order to employ it advantageously, he observed, we should indiscriminately have daily access to it—and not only the profession in this country, but strangers from other countries should be permitted to examine and make drawings from the preparations in this splendid collection. Instead of this, it is well known to be locked up eight months in the year, and when open, the restrictions are such as to render its exhibition comparatively useless.

Mr. Lawrence averred that, in his intercourse with the profession, he found but one sentiment prevail—diapprobation of the College—although there might, and doubtless would be, many opinions as to the remedy. Mr. L. satirized with some humour, the nice distinctions which are made between the *pure* surgeons and the general practitioners, remarking, that although the former class held themselves aloof from the latter, as things unclean, yet there was one way of bringing them into close contact and harmonious co-operation—namely, through the medium of a consultation, or in other words, a fee. On such occasions, the impure surgeon is sure to have a most polite and cordial reception from the pure!

Mr. L. in conclusion, made a solemn appeal to the good sense of the assembly, exhorting them to moderation and decorum, as the surest means of attaining their object—a moderation and decorum which certainly was not always strictly observed in the course of the debates, as the chairman himself was forced to admit. He wisely conjured them "*to bear in mind, that the eyes of the profession and of the public were on the proceedings of this evening, and that if they were not conducted so as to secure general approbation, they would not only fail of the effect of producing a remedy, but they would give additional force and duration to their grievances.*"

Mr. Tyrrel rose and observed, that the present constitution of the Royal College of Surgeons is the same as when it was incorporated with the Barbers—and is not now so good as that of the knights of the razor. The latter have the privilege of excluding members from practising on the chins of his Majesty's subjects, whereas any man may practise surgery under the very Portals of the College, with perfect impunity, however unfit for such practice. He adverted to the shameful restriction on the museum—the defect in the constitution of the College, where they have the power of electing themselves, and doing what they please with the funds. Mr. T. read the first resolution, purporting "that the public and the members of the surgical profession may justly complain that the science of surgery has not been advanced, nor its practitioners benefited, either by the late corporation, or the present Royal College of Surgeons." This was seconded by Mr. Wardrop, and carried unanimously.

Mr. Paty proposed the second resolution, which went to accuse the court of examiners of slurring over the examination of candidates for diplomas, from thirst of gain, and thereby permitting improper persons to have their names registered on the list of the College, many of them being now regular advertising quacks. Carried *nem. con.*

Mr. Kingdon made some pertinent observations on the regulation of the College passed in 1823, prescribing three winter courses of anatomical lectures, thus rendering null the certificates of summer courses, hitherto admitted. It is evident that this regulation was an infringement on the property of those teachers who were in the habit of giving summer courses of lectures, and injurious to those students who wished to profit by the leisure of summer, in increasing their anatomical knowledge. As the *tendency* of this regulation (the *design* of it being left out of the question) was to increase the private interests of the framers of it, Mr. K. was convinced, that such consideration would induce the legislators of the College to abrogate a law which was capable of being construed in a manner injurious to their private characters. Mr. K. therefore moved a resolution, that the said regulation of 1823, contained provisions of the most oppressive character, injurious to the rights and property of individuals, and calculated to increase the expence and difficulty of acquiring surgical knowledge, while it served the private interests of the ten examiners, by whom the regulation was made. *Carried unanimously.*

Some questions were now asked as to the authors of the advertisements, calling the meeting; when Mr. Wakley (the Editor of the Lancet) acknowledged himself as one.

Mr. MACILWAIN moved a resolution, the gist of which, as well as of his preliminary observations, was to stigmatise the bye-law, now so famous, which limits chirurgical tuition to certain schools and their protégés. This was seconded by Mr. Key, and carried of course.

Mr. WELBANK moved, as a corollary of the preceding resolution, that the bye-law in question, cast an unmerited stigma upon the surgeons of the various provincial hospitals, who are in no respect inferior to their brethren in schools so much favoured by the College. *Carried.*

MR. LLOYD addressed the meeting respecting the museum, through which gentlemen were shewn, somewhat in the same manner that they are shewn round the cribs of wild beasts in Exeter 'Change. (*Laughter.*) Mr. L. complained that no catalogue had yet been made of the preparations, by means of which, they could be seen with advantage. The large mass of valuable manuscripts, too, left by John Hunter, has been burnt by one of the Council, to the consternation of Lincoln's Inn, as the flames ascended into the air, and induced the fire-men to rush into the College, thinking it was in a state of inflammation! Who the incendiary was, on this occasion, we do not hold ourselves bound to pronounce. Report says, that when the fire-men broke in, they found only ONE of the Council at HOME, that evening. Mr. Lloyd complained, also, that access to the library was denied to all but the Council. He moved a resolution, that the museum has been so managed as to be of little or no public utility—that it is inaccessible to the members during eight months of the year, and open only twice a week, for four hours, during the remaining four months—that the library is without a librarian or catalogue—and, consequently, that both the museum and library are comparatively useless to the members of the College.—*Carried nem. con.*

Mr. WAKEFIELD moved the next resolution, which was hardly worthy of the serious attention of the assembly—namely, that the members in general have just reason to complain of being obliged to enter the theatre of the College through the door at the back of the building, whilst the Council and their personal friends have entrance by the portico in Lincoln's-Inn-Fields. Mr. Leese seconded the motion, and enlarged upon the grievance in question, when the resolution was carried unanimously.

MR. WIGAN got up, and in a short but rather pithy speech, observed that, in respect to the grievances complained of that night, there appeared to be but one sentiment—that of indignation. But as to the remedy, there might, and no doubt would be, some diversity of opinion. He begged to remind the audience that the proceedings of that evening would be much more likely to prove successful, by carrying with them the current of public feeling, if the remedy projected, exhibited no character of violence. He therefore, proposed that "a Committee be appointed to prepare, for presentation to the Council of the College, a remonstrance, grounded on the foregoing resolutions, expressing in firm, but respectful language, the dissatisfaction with which this Meeting contemplates the various matters enumerated in these Resolutions, and requesting that such measures may be adopted, to remedy the grievances complained of, as the Court may deem advisable."

Here Mr. Wakley, the editor of the Lancet, made a long and a vehement appeal to the passions, rather than to the reason, of the audience, during which harrangue, there was such a mixture of approbation, laughter, and hissing, that Mr. Lawrence threatened to leave the chair. No measure, but that of uprooting the Constitution of the College, would serve Mr. Wakley.—He, therefore, moved a long amendment, the gist of which was, that the Charter of the College was unconstitutional, by conferring on the Council and Court the privilege of electing those who are to be their colleagues in office—that this is the root of all the evils and grievances—that Parliament be petitioned to enquire into said abuses and mal-practices, with the view of ultimately obtaining from His Majesty a new charter, which shall provide that the officers of the College be *annually* chosen by the Members, so that *each Member* may have a voice in the election of said officers, &c.

Several gentlemen now spoke for, and against, this direct appeal to Parliament, and the sober and temperate party were likely to carry the resolution of remonstrance to the College, when Mr. Wakley made another, and a still more inflammatory, speech, in which every nerve was exerted to excite indignation against the officers of the College—and with too much effect; for, notwithstanding the most unequivocal expression of the chairman's own sentiments in favour of Mr. Wigan's motion for a remonstrance, the amendment of Mr. Wakley was carried by a majority of about three to one. This, of course, terminated the string of resolutions, and it only remained to appoint a committee for preparing the petition to Parliament:—But here a scene occurred which the *veracious* LANCET, and its pure and patriotic conductors have *most carefully suppressed,* though witnessed by eight—or, as some say, twelve hundred individuals! Elated by the bursts of applause which attended Mr. Wakley's invectives against the College—and, mistaking approbation of the *measure* for approbation of the *mover,* a person came forward and proposed that the *Editor of the* LANCET *should, as a matter of course, be nominated on the Committee, in testimony of what they owed to that excellent and independent Journal, for bringing together this numerous assembly.* But here vanity and ambition, by overshooting the mark, were destined to experience a most mortifying rebuff—and the vessel which steered for the harbour under a full gale of apparent popularity, was wrecked at the very entrance of the port! Instead of the expected peal of plaudits, this proposal was met by hisses and cries of no, no, from all parts of the house—and, although the *gentleman* sprang on the hustings and repeatedly attempted to speechify, his voice was drowned at each attempt—till the editor of the Lancet, clearly perceiving the force of this counter-current, put an end to the contest by declaring that *his avocations prevented him from accepting the office of Committee-man!**

A committee of 21 persons being appointed, and thanks being voted to the Chairman for his able and impartial conduct, the meeting was adjourned till Saturday, the 4th March, at the same hour and place.†

---

———————————————— horum

Nunquam Ego optârim pauperrimus esse malorum.

† This second meeting was much more thinly attended than the preceding. It appears that the Committee had forwarded to the College a copy of the resolutions passed at the first meeting, requesting to know how far *the College would join them in a petition to Parliament for a new Charter!* This is one of the best jokes we ever recollect to have heard in the whole

It now remains for us to make some brief remarks on the causes, conduct, and probable consequences of this meeting, conscious that our observations, however unsatisfactory they may be to either of the contending parties, will, in the end, be acknowledged as impartial, and dictated by a sincere wish for the welfare of the medical profession.

In the first place, we think it would be extremely difficult for the warmest advocates of "things that be," to convince the unprejudiced part of the community of the non-existence of evil, or, at least, of negative good, in some of the present laws, and some parts of the present administration of the College of Surgeons. The obnoxious bye-law, to which we have so often alluded, is absolutely indefensible—at least, we shall consider it as such, till we hear some arguments in its favour. That the Museum should be thrown more open, and, consequently, rendered more available for the purposes of physiological and pathological science, is as evident as the sun at noon-day. That those who enter the College by the portico of Machaon and Podalirius, with a pass-port of twenty guineas in their hands, for the certificate of an examination within the sacred walls, should be ever afterwards admitted by the same channel, is a wish so reasonable, though apparently so childish, that we marvel much the invidious distinction of different doors to the temple of science should ever have been contemplated by men of a liberal and enlightened profession. Why keep up a paltry distinction which can either flatter pride on one side of the College, or mortify it on the other?

That the magnificent revenue of the College, derived, as it is, from those who *voluntarily* go there for diplomas, might support a more extended course of instruction at the College, is, we think, a very reasonable opinion of its members, and one which deserves serious consideration from the Council.

The grievance, which we consider as one of great magnitude—and which is common to the Council as well as to the Members of the College, is the want of power in the College to prohibit the practice of irregular surgeons. This was scarcely touched upon in the deliberations of the assembly. It is

---

course of our sojourn in this world. The reply of the College was laconic, but to the point. They were always ready to pay due attention to the suggestion of Members, "*when directed to the common weal,*" but the Council could not hold communication with persons, whose avowed object was the subversion of their charter. It was then proposed that the petition should be laid before a Parliamentary counsel for revision. Orator Wakley then rose, "and (we quote from the Sunday Times of March 5th) attempted to address the meeting, but the groans and hisses were so loud that it was impossible to hear what he said." The Chairman at length interposed, and the Orator was allowed to have his say, and receive considerable plaudits from the junior portion of the audience.* Mr. Tyrrel remodelled his resolution into the following terms—"*that the Committee be at liberty generally to take such steps as they may think fit for the attainment of the object of the meeting, viz. the remedying the abuses now existing in the Royal College of Surgeons, &c.*" According to this resolution the Committee has the power of following up the original resolution of memorialing the College itself for redress of grievances, as first proposed by Mr. Wigan—and verily we think they will act wisely in taking this more peaceable and more regular step, before they address themselves to the great National Council.

* It should be recollected that this assembly had it all their own way. They, almost all, went to Freemasons' Tavern predetermined as to their sentiments and conduct. All the upper classes held back—well knowing the diabolical character of certain agents who were at work, to lead them into error and intemperance—agents who had nothing but self-interest at bottom.

well known that Parliament refused to interfere in this business—and this induces us to think there is little chance of redress to be obtained from Parliament on future occasions. But if the College have no power to prohibit unqualified practitioners, they surely might devise means of exposing those of their own members who disgrace the profession by open quackery. The College of Physicians have struck off unworthy members from their lists—and cannot the College of Surgeons do the same?

We have now passed in review the grievances complained of by the Members of the College, and, we are sorry to confess our conviction of their existence, in a greater or less degree. We are next to glance at the conduct of the assemblage which took place at the Freemasons' Tavern.

In the first place, we must observe, that the meeting was open to all—and, consequently, that a great number were present who had no right to express any opinion—and still less to give any vote on the different resolutions proposed. This must, in a great degree at least, have vitiated the result. We had ocular demonstration of hands and voices raised, on several occasions, where no right existed in the individuals for such interference. That turbulence and disorder appeared in the meeting, we have the President's own testimony, who, more than once, threatened to leave the chair. That *some* of the orators endeavoured, by all possible means, to work on the passions rather than on the reasoning-powers of the assembly, was clearly manifest—and that, hurried along by their passions, the majority of juniors overpowered the temperate and the wiser minority of seniors, is, to our minds, most evident. That the decision, in respect to the main proposition, was carried against the sense of the Chairman and of the senior members present, admits of no question—and whether or not they acted wisely in so doing, will be seen in the sequel. In short, there never was an assemblage seen, in which the turbulent passions of a demagogue more completely triumphed over the sound sense and sober reasoning of the audience!

The last thing which we have to consider is, the probable consequence of this meeting and the measures taken on the occasion. Will the Parliament entertain the petition, and order a committee of enquiry into the alledged abuses of the College? We cannot believe they will; because the immediate object of the petition is not the reform of abuses, but the abrogation of the charter. There is no proof offered—there can be no proof offered, that all the abuses and grievances complained of, might not be remedied without such abrogation. We all know, indeed, that unlimited power and security are, what may be termed predisposing causes to abuse, but it does not necessarily follow that the one shall lead to the other. But it may be said, why apply for a redress of grievances rather than the removal of their causes? We answer that the Charter of the College cannot—should not be abrogated. The mode of election in the College Charter is more or less identified with that of all other colleges, literary or scientific, in the kingdom, and Parliament will probably not feel inclined to open the door to such an innovation, on the prayer of the members in question. That College which comes nearest to the popular constitution now sought, is the College of Dublin; but even there, the *elective franchise* is confined to those who actually reside in Dublin, and who are obliged to pay a heavy fine for the power of voting. Again. Is it certain that a purely popular representation in the College of Surgeons would be attended with all the good expected? Of this we have no means of judging. Many constitutions look fine on paper, which make a sad figure in practice. We can only judge by analogy; and, if we take the late meetings as examples, the new order of things in Lincoln's-Inn-Fields might chance to be governed by violent men, and characterized by intemperate measures! It would certainly be better to be ruled by 12 tyrants (if tyrants) than by 1200! But is it likely, we ask, that the Legislature, which has already refused to interfere on points of the most vital interest to the whole medical profession, and to the whole community at large, (the difficulties which beset the study of anatomy, for example, and the free permission for the most ignorant men to practise surgery) is it likely, we repeat, that it will interfere at the solicitation of a portion of the Surgeons of London, whose direct application to Parliament (with the view of abrogating the charter) was carried, through the instrumentality of one who is a disgrace to the medical profession and who is banished from the practice of it, against the advice and opinion of all the senior and influential men of a public meeting, including their own chairman, in favour of a prior remonstrance to the College itself?—This is a question which time will answer.

In the mean while we venture to believe, that public opinion will do more for the redress of grievances than the national senate. We think it cannot be denied, that the alledged grievances exist in degree, and there is good sense and honorable feeling enough in the ruling men of the College, to see the propriety of redressing them.* It is probable, however, that the resolution to carry their complaints at once before Parliament, (if persisted in) will delay any measure of reformation in the College, till the fate of the petition be known, since any concession at the present moment, would look like the effect of intimidation. Whatever be the fate of the parliamentary appeal, we are convinced that the agitation of the question will be attended with ultimate good effects—though not to the extent anticipated by the ardent minds of the younger branches of the surgical profession. The influence of public Colleges on the faculty at large we do not deny—but it is far less than is generally supposed—infinitely less than that of public opinion as conveyed through the medium of the press. Our final advice would be—moderation on the part of the Members, and conciliating redress on the part of the Council of the College. An abrogation of the charter we consider unattainable by the present means—and if attained, of very doubtful benefit, and probably of much detriment, to the community at large.

---

Before quitting this subject, we beg to express our full conviction of the truth of the ancient doctrine of transmigration—and that the soul of NOAH BRIGGS, after various incarnations, has at length animated the renowned and disinterested champion of medical reform—*Orator Wakley*. Be it known to the reader, that the said Noah Briggs did, in the time of Oliver Cromwell, petition parliament for a reform in the abuses of the Colleges of his day—holding forth in his "*Matæotechnia*, wherein is dissected the errors, ignorance, and supinities of the schools," the very same argumentation, declamation, and defamation, which was thundered forth through Freemasons' Hall, by the Editor of the Lancet. An extract from this curious petition to the Rump Parliament, may not be void of amusement.

"How ill disposed are those few Colleges in this land, that should be collateral or subservient to this designe? Or wherein do they contribute to the promotion or discovery of science? Where have we Professors and Lectures? Or how cold and lazily are they read, and carelessly followed? Where is there any examination and consecution of experiments? Encou-

---

* We understand that a memorial, signed by all those of rank, talent, and influence in the Surgical Society of the metropolis, has just gone into the College—supporting and approving of the Council, but praying for a modification of certain regulations, &c. This is as it should be.

ragement to a new world of science?—Where have we constant readings upon either quick or dead anatomies?—Where a review of the old rubbish that has pestered the temple of knowledge—of that farraginous syndrome of knaves and fools huddled together, whose habilities are not tempered to enlarge the territories of knowledge—whose unqualified intellectuals are unable to rectify the errors of their reason, and are only fit to maintain error and their present places—who know only what has been hammered into them by ill methods and thumping teachers—*while those who have a greater vivacity of more sublimed spirits, and understanding, far above theirs that taught them what they know, are dejected with obloquie, or cold encouragement!* In their names I shall not, for neither friend nor foe, conceal what the general murmur is—that science is become a deformed and ill-favoured MEDUSA, with her tresses full of adders—that there is no public encouragement given to the friends of science, who are evidently constellated to study, and *love vertue and learning for itself, not for lucre, or any other end but the good of their country, &c.*" Noah Briggs' petition to Parliament, p. 8.

Among the specific redresses which are prayed for by Noah Briggs, one or two are somewhat curious at the present day.

"*Secondly*, That you would call forth *some*, and enable them with authority to see the College of ——— reformed, and laboriously rummaged in her stupendous bulk of learning, that so the great ocean of universal knowledge may run pure and fair in this nation—that so our youth may not be trained up in error and the destruction of men."—* * * *

"*Fourthly.* That the Temple of Esculapius may, like that of Janus, with his *two controversial faces*, be set wide open to all, without distinction." p. 11.

From these extracts it will be seen that there is nothing *new*, even in our grievances—and quite as little novelty in the orations of our popular regenerators.

